# A feasibility study of wireless inductively coupled surface coil for MR-guided high-intensity focused ultrasound ablation of rodents on clinical MRI systems

**DOI:** 10.1038/s41598-022-26452-z

**Published:** 2022-12-19

**Authors:** Chien-Feng Judith Huang, Win-Li Lin, San-Chao Hwang, Ching Yao, Hsu Chang, Yung-Yaw Chen, Li-Wei Kuo

**Affiliations:** 1 Department of Biomedical Engineering, National Taiwan University, Taipei, 100233 Taiwan; 2 Institute of Biomedical Engineering and Nanomedicine, National Health Research Institutes, Miaoli, 35053 Taiwan; 3 MBInsight Technology Corporation, New Taipei City, 236658 Taiwan; 4 Department of Electrical Engineering, National Taiwan University, Taipei, 100233 Taiwan; 5 Institute of Medical Device and Imaging, National Taiwan University, Taipei, 100233 Taiwan

**Keywords:** Preclinical research, Biomedical engineering

## Abstract

Recently, to conduct preclinical imaging research on clinical MRI systems has become an attractive alternative to researchers due to its wide availability, cost, and translational application to clinical human studies when compared to dedicated small animal, high-field preclinical MRI. However, insufficient signal-to-noise ratio (SNR) significantly degrades the applicability of those applications which require high SNR, e.g. magnetic resonance guided high-intensity focused ultrasound (MRgHIFU) treatment. This study introduces a wireless inductively coupled surface (WICS) coil design used on a clinical 3 T MRI system for MRgHIFU ablation. To evaluate the SNR improvement and temperature accuracy of WICS coil, the ex vivo experiments were performed on the pork tenderloins (*n* = 7) and the hind legs of deceased Sprague–Dawley rats (*n* = 5). To demonstrate the feasibility, the in vivo experiments were performed on the hind leg of Sprague–Dawley rat (*n* = 1). For all experiments, temperature measurements were performed before and during HIFU ablation. Temperature curves with and without WICS coil were compared to evaluate the temperature precision in ex vivo experiments. The use of WICS coil improves the temperature accuracy from 0.85 to 0.14 °C, demonstrating the feasibility of performing small animal MRgHIFU experiments using clinical 3 T MRI system with WICS coil.

## Introduction

High-intensity focused ultrasound (HIFU) is a noninvasive thermal ablation method, which can transmit thermal energy to target tissues. During HIFU power deposition, the noninvasive thermal mapping capabilities of magnetic resonance imaging (MRI) play a crucial role in monitoring tissue temperatures to avoid unwanted damage to neighboring healthy tissue. In recent years, magnetic resonance guided HIFU (MRgHIFU) therapy has been increasingly used to treat several kinds of diseases^1^, such as uterine fibroids^2,3^, brain tumors^4^, and essential tremor^5^. Compared with conventional surgery, MRgHIFU has become an alternative treatment option with several advantages, such as lower cost and shorter recovery time.

Preclinical research of MRgHIFU treatment is highly beneficial to the translational applications because it forms the foundation for future clinical studies. For preclinical research, rodent models are common choices because they can be used to explore the underlying mechanisms and evaluate the therapeutic approaches for diseases. To conduct preclinical researches, dedicated small-animal and high-field (> 4.7 T) MRI systems are well suited in rodents due to their capability of providing better signal-to-noise ratio (SNR) and image quality. However, high-field MRI systems dedicated for small animal use are relatively expensive, of limited availability, and has markedly different settings from clinical MRI (including pulse sequence, magnetic field strength and hardware settings)^6,7^. Compared to dedicated high-field MRI systems, clinical MRI systems are widely available and the developed techniques can be easily translated or applied to clinical human studies. Thus, conducting preclinical studies on clinical MRI systems are an attractive option to researchers who are interested in translational applications.

Despite the advantages of conducting preclinical studies on clinical MRI systems, the relatively low SNR for imaging rodent models on clinical MRI systems usually limits the translational capability^7,8^. To improve local SNR, previous studies used the dedicated receive-only surface coils or clinical flex MRI coils placed close to the region of interest (ROI), localizing the sensitive region with the ROI and diminishing the total noise received^9–15^. However, the coil size and shape of either the dedicated receive-only surface coils or the clinical flex MRI coils are typically designed for specific purpose by the vendors and may not be suitable for the MRgHIFU use. In addition, the connection between the MRI mechanism and surface coil/clinical flex MRI coil may be interfered by necessary transducer movements during MRgHIFU treatment. Therefore, it is emergently to design some other approaches to improve the SNR for MRgHIFU use on clinical MRI systems.

Wireless inductively coupled surface coil (WICS coil) is another option which has the same purpose of a general surface coil but circumvents the drawbacks mentioned previously. Unlike the custom-made surface coil, WICS coil is simple to implement—an improved SNR can be acquired by placing a wireless resonance L-C circuit with matching frequency in the current MR setup. Because of its ease of implementation, the WICS coil has been used in MR guided brain biopsy^16^, patient motion correction^17^, stents^18^ and catheters visualization^19^. Furthermore, the WICS coil design has no physical connection between the MRI system and coil, preventing the limitation of a physical connection hampering transducer movements during MRgHIFU treatment. By improving local SNR, the WICS coil could also improve temperature accuracy within the heated treatment region and has the potential to enhance performance in MRgHIFU treatment.

The goal of this study was to evaluate the feasibility of the WICS coil on clinical MRI systems to perform MRgHIFU ablation for small volume objects, via the experimental demonstration on ex vivo pork tenderloins, ex vivo rats and in vivo rat. We aimed to enhance temperature accuracy from a few degrees to less than 1 °C (the ideal temperature accuracy of thermal therapy)^20^, exploring its capability of being the alternative to dedicated high-field MRI or custom surface coils for preclinical studies.

### Theory

**Figure 1:**
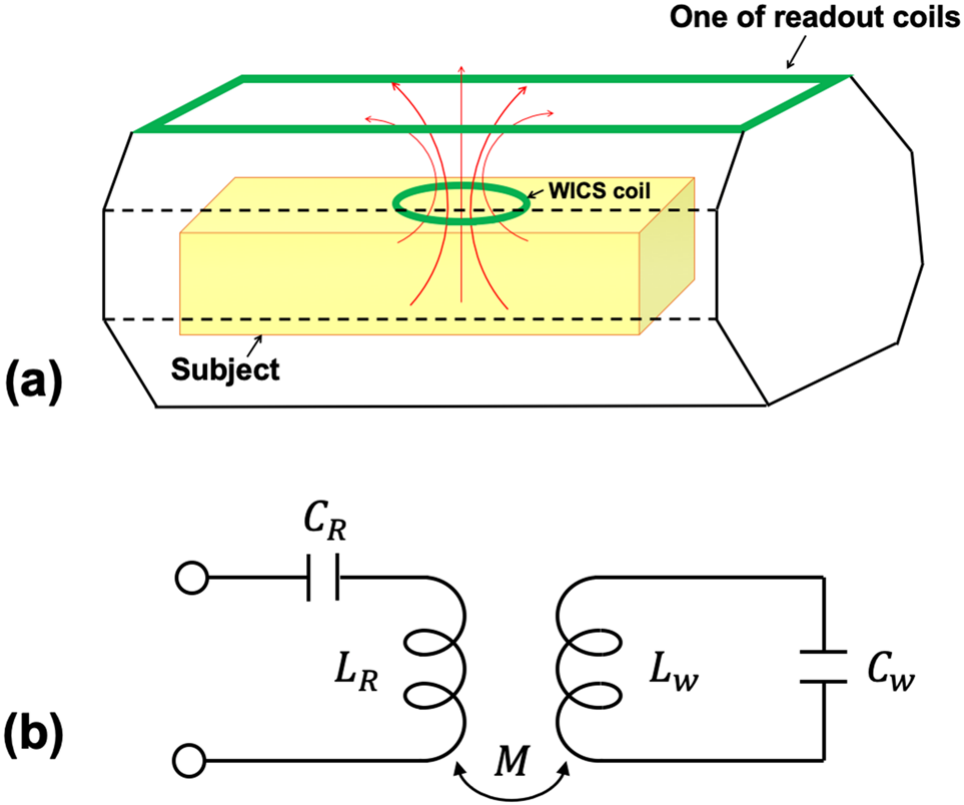
(**a**) An illustration for the operation of mutually inductively coils. Red arrows indicate the flux-linkage between WICS coil and readout coil. The signals around WICS coil can be transmitted via flux-linkage with diminishing amount of noise, achieving SNR improvement. (**b**) The equivalent circuit used in mutually inductive coupling. The values, ${L}_{R}$, ${C}_{R}$ and ${L}_{w}$, ${C}_{w}$ are inductance and capacitance of readout coil and WICS coil, respectively.

The concept of WICS coil is based on the principle of mutually inductive coupling^21–25^. The scheme contains a WICS coil and a readout coil that are resonated to Larmor frequency of the MR scanner, as illustrated in Fig. 1(a). The WICS coil placed in ROI and readout coil were connected to the RF receiver of the MRI, sharing magnetic field and induce voltages with each other. The flux linkage between the two coils was generated, thus the signal around WICS coil can be efficiently and wirelessly transferred to the readout coil. Smaller sensitive region of WICS coil brings about diminishing the amount of receiving noise and further improves the SNR of ROI.

The equivalent circuit for “flux-linkage” coupling is shown in Fig. 1(b). The readout coil of inductance ${L}_{R}$ and capacitance ${C}_{R}$ is coupled to the WICS coil of inductance ${L}_{w}$ tuned with capacitance ${C}_{w}$ via generating mutual inductance M. The mutual inductance, M, is given by

1 $$ M = k\sqrt {L_{R} L_{w} } $$

where, M is the mutual inductance between ${L}_{R}$ and ${L}_{w}$ and k is coupling coefficient.

## Methods

### WICS coil design

**Figure 2:**
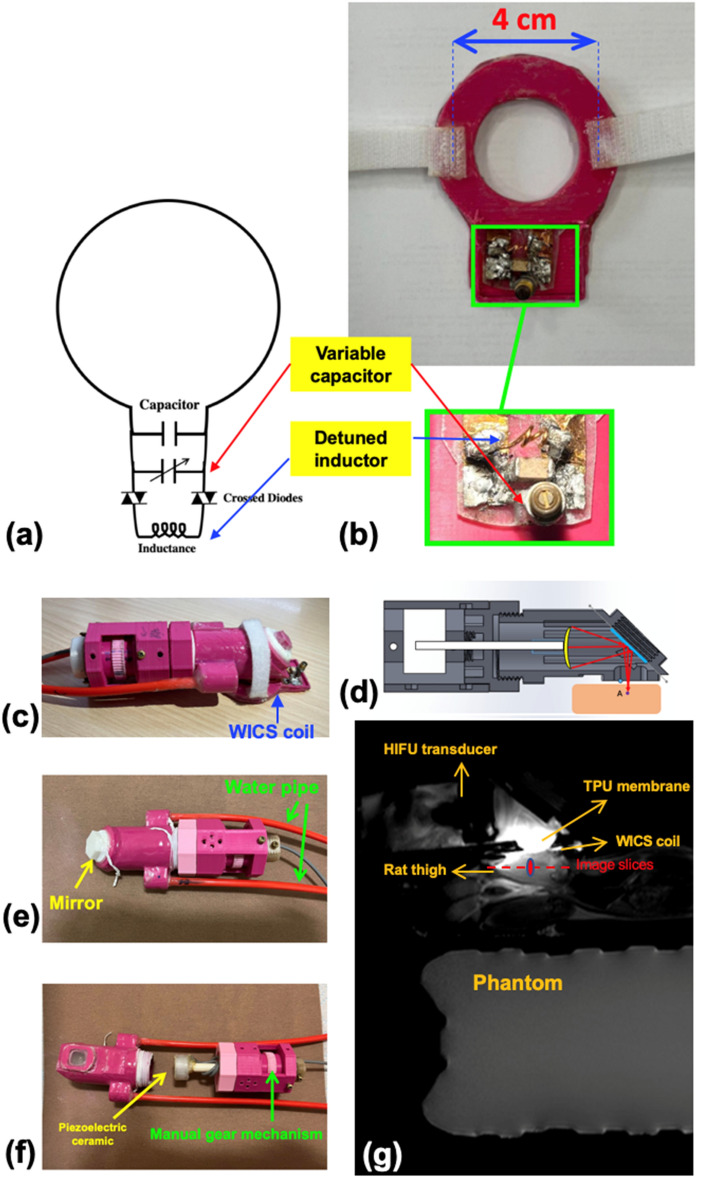
(**a**) Circuit diagram and (**b**) photograph of the WICS coil. The shallow shape of the WICS coil enables a close contact with the HIFU transducer during HIFU ablation. (**c**) Photograph of WICS coil implemented in the HIFU transducer. Increased SNR is beneficial to improve the temperature accuracy of MR thermometry. To enhance the SNR of the focal region, the home-made WICS coil was positioned to surround the HIFU transducer inside the MRI scanner. (**d**) Schematic and (**e**, **f**) photographs of the home-made reflecting transducer. (**g**) Illustration of the experimental setup. The HIFU transducer was placed on a rat thigh, and the glass in front of the piezoelectric ceramic reflects the HIFU power into the rat thigh.

The circuit design and prototype of the WICS coil are shown in Figs. 2(a and b), respectively. The resonance frequency of WICS coil was adjusted to match the Larmor frequency of the 3 T MRI system (Achieva, Philips Healthcare, Amsterdam, Netherlands) at 127.8 MHz. In our study, the total bandwidth is 1.9 MHz (i.e., a plus or minus 0.95 MHz). According to our measurement, the quality factor of the WICS coil is 67.7 and its full width at half maximum is 1.90 MHz. Because of this, a coil frequency centered at 127.8 MHz within a range of 1.90 MHz is acceptable. The WICS coil consists of a copper circular wire (diameter of 4 cm), a 16-pF capacitor (Voltronics, USA), a variable capacitor (NMKM10HV, Voltronics, USA) and a detune circuit. We used a network analyzer to check the resonance frequency of WICS coil and fine-tune the coil frequency during the experiment setup to ensure that frequency variation stays within the acceptable error range. To ensure signal homogeneity and the “receive-only coil” nature of the WICS coil, two passively crossed diodes (UM9989, Microsemi) were used to decouple the resonance circuit. Theoretically, it is sufficient to use only a pair of crossed diodes, but we decided to use two pairs of crossed diodes to increase its withstand surge voltage. When high-power RF-pulses are applied, the WICS coil is simultaneously detuned to prevent coupling effects in the transmit mode of the MR system^26^. For WICS coil decoupling validation, we put a double loop probe near the WICS coil to check the S21 difference while the WICS coil at on-resonance or off-resonance states, by applying a DC voltage (5 V) to turn on/off the cross-diodes. The loops of probe have the same diameter of 6 mm and are overlapped for completely decoupling each other. Note that the amount of decoupling in our case was 20 dB according to decoupling validation method described above.

The WICS coil was mounted on a 3D-printed hollow shell (Polylactic Acid). The diameter of the WICS coil is 4 cm. The proposed shell shape was designed to support the MRgHIFU setup so that the HIFU transducer is surrounded by the WICS coil, ensuring higher image quality in the heated region [Fig. 2c]^21^.

### Theory of MR-based temperature measurement

The MR temperature measurements were analyzed based on the proton resonance frequency (PRF) shift method as follows^27–30^:

2 $$ \Delta T = \frac{\emptyset \left( t \right) - \emptyset \left( 0 \right)}{{\gamma B_{0} \alpha TE}} $$

where, $\gamma$ is the gyromagnetic ratio,${B}_{0}$ is the amplitude of the static field,$\alpha (-0.01\mathrm{ ppm}/ ^\circ \mathrm{C}$) is the temperature-dependent coefficient for tissue, TE is the echo time of the sequence, $\boldsymbol{\varnothing }\left({\varvec{t}}\right)$ is the phase at time t, $\boldsymbol{\varnothing }(0)$ is the phase before heating.

The SNR affects the accuracy of $\Delta \mathbf{T}$ calculated from Eq. (2). Temperature accuracy (${\sigma }_{T}$) is inversely proportional to the SNR of the magnitude image^31^:

3 $$ \sigma_{T} \sim \frac{1}{SNR \times TE} $$

Thus, the SNR is a crucial factor affecting temperature accuracy. A higher SNR results in improved temperature accuracy. Note that temperature accuracy in this study indicates how close temperature values obtained from MR thermometry are to actual temperature values. Improved temperature accuracy indicates better temperature precision.

### Reflecting HIFU transducer

A home-made reflecting HIFU transducer was designed for this study. The schematic drawing and prototype of the transducer are shown in Fig. 2(d,e,f). A 4-MHz piezoelectric ceramic was mounted inside a 3D-printed shell (Polylactic Acid) and a glass piece was placed 45° in front of the piezoelectric ceramic to reflect the HIFU power, changing the direction of the HIFU power. The HIFU power then passes through a thermoplastic urethane (TPU) membrane to reach the target tissue. A gear structure located behind the piezoelectric ceramic (at the tail-end of the transducer) can be turned manually to move horizontal bar attached to the piezoelectric ceramic, changing the distance between the piezoelectric ceramic and glass piece, which then changes the depth of the HIFU focal zone. Two water pipes were used to circulate water inside the transducer to dissipate extra heat produced.

### MR thermal mapping validation study

To verify the accuracy of MR thermometry, measurement of the actual temperature change by using a fiber-optic thermometer was performed to compare with MR thermometry results. However, this validation method is impractical in HIFU ablation experiments because the HIFU transmission interferes with the fiber-optic thermometer. Thus, we used a hot water pipe to heat the pork tenderloin (instead of HIFU transmission) to validate MR thermometry results during heating. A water pipe filled with circulating hot water (70 °C was pierced through the pork tenderloin, and a fiber optic thermometer used to record the actual temperature was placed next to the water pipe.

**Figure 3:**
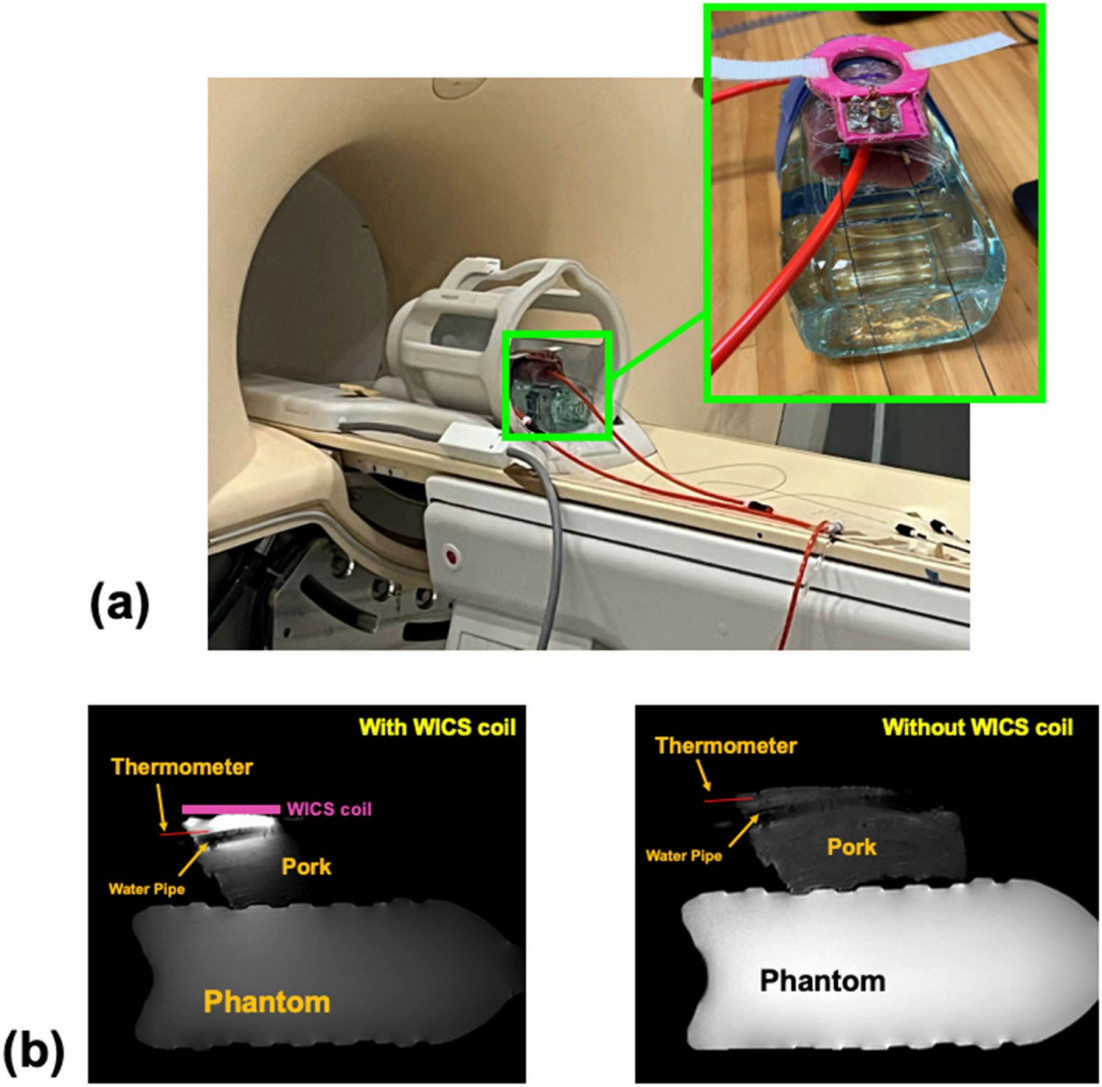
(**a**) Photograph and (**b**) experimental setup of validation study. A water pipe filled with circulating hot water was pierced through the pork tenderloin. Two fiberoptic thermometers were inserted to measure actual temperature inside the pork tenderloin to validate MR thermometry.

Experimental setup is shown in Fig. 3. The experimental setup and anatomical localization scan took approximately 20 min. The T2-weighted images and temperature mapping described in this paper were acquired with turbo spin echo and gradient echo pulse sequences, respectively. The sequence parameters applied for T2-weighted images used for positioning were field-of-view (FOV) = 230 × 184 mm^2^, matrix size = 400 × 256, repetition time (TR)/TE = 3000/80 ms. Sagittal sections were used for positioning. After T2 images were acquired, MRI temperature measurements without and with heating via hot water circulation were performed with parameters: TR of 16 ms, TE of 12 ms, flip angle of 30°, matrix size of 128 × 128, FOV of 128 × 128 mm^2^, slice thickness of 3 mm, and slice number of 4. The first set of scans were performed without using the WICS coil. The scan protocol and experiment were performed after placing the WICS coil on the pork tenderloin to improve the SNR of MR thermal mapping.

### MRgHIFU system setup

**Figure 4:**
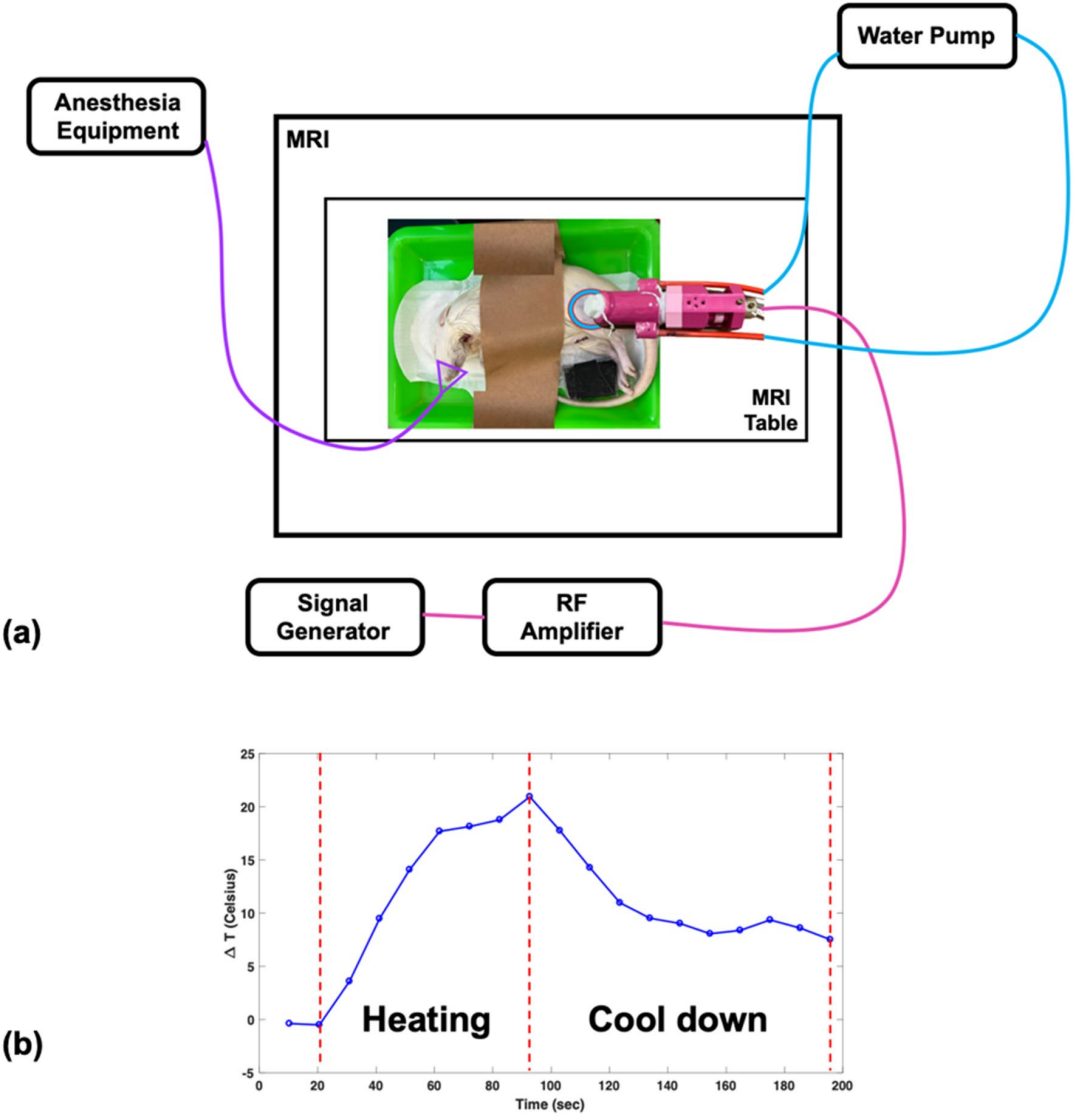
(**a**) Setup of HIFU experiment. The reflecting HIFU transducer, derived from the RF amplifier, was placed above the rat thigh or pork tenderloin, and the WICS coil was mounted around the transducer. (**b**) The temperature response shows the heating procedure of the HIFU experiment. HIFU power was turned on for an ultrasonic dose of 30 w/60 s then turned off for tissue cool-down until end of scanning.

The MRgHIFU system design and its experimental setup are shown in Figs. 2(g) and 4(a). A home-made reflecting HIFU transducer (outlined in the “Reflecting HIFU transducer” subsection) was placed above the rat thigh or pork tenderloin. As mentioned in the previous section, the HIFU transducer uses a glass piece to reflect HIFU power, which then passes through a TPU membrane and converges into a focus zone to ablate the rat thigh or pork tenderloin. A phantom consisting of 1.25 g NiSO_4_ × 6H_2_O and 5 g NaCl per 1000 g H_2_O was placed below the rat thigh or pork tenderloin and the head or body coil was used for imaging small sample. In the in vivo experiment, mask cones were placed over the rat’s face for inhalation anesthesia. The WICS coil (described in the “WICS design” subsection) was mounted around the HIFU transducer to improve image quality around the ablation point. The broadband RF power amplifier (0.01 ~ 230 MHz, 100 W, EMPOWER), used for the HIFU transducer, was setup outside the MR scanning room. Note that the same experiment without WICS coil was performed to compare the results.

All T2-weighted images were applied with parameters of FOV = 230 × 184 mm^2^, matrix size = 400 × 256, TR/TE = 3000/80 ms. Sagittal sections were used for positioning and SNR comparison. All temperature mapping were applied with parameters of TR = 16 ms, TE = 12 ms, flip angle = 30 $^\circ$, matrix 128 × 128, FOV = 128 × 128 mm^2^, slice thickness = 3 mm, and slice number = 4 in coronal sections. All MR raw data was reconstructed and analyzed offline in Matlab R2016b (MathWorks, MA, USA).

### Ex vivo experiments

Ex vivo experiments were separated into two parts, including a temperature measurement comparison study and a SNR comparison study. The temperature measurement comparison study was performed on pork tenderloins (250–350 g, *n* = 7) and hind legs of deceased rats (250–350 g, *n* = 5), using a clinically used body coil as the transmit coil and an 8-channel human head coil as the receiving coil.

1. Pork tenderloins: Pork tenderloins were purchased from the local supermarket and placed in the scan room at room temperature for two hours before the experiment. The experimental setup is shown in Fig. 2(g) and described above. The experimental setup and anatomical localization scan took approximately 20 min. T2-weighted images were used for positioning. After T2 images were acquired, thermal mapping both without and with HIFU ablation was performed using spoiled GRE pulse sequences. After scanning for 30 s, HIFU ablation was applied at 30 W for 60 s, and then HIFU power was turned off to allow the tissue to cool-down. Ablation procedure is shown in Fig. 4(b).
2. Rat: Deceased rats were stored frozen. Before the experiment, deceased rats were soaked in room temperature water to thaw for two hours and placed in the scan room at room temperature for one hour. Fur on the rat thighs were shaved to improve deposition of ultrasound beam into the tissue. Rats were placed in the right recumbent position without WICS coil to obtain reference images. Rats were then moved to the left recumbent position, and the WICS coil was placed on the right hind thigh inside the receive coil. The experimental setup, process and MRI protocols are the same as those mentioned in the “Pork tenderloins” subsection.

The SNR comparison study was performed on the pork tenderloins (250–350 g, *n* = 7) and hind legs of deceased rats (250–350 g, *n* = 5), using the body coil as the transmitting coil and receiving coil. The experiment setup and process are the same as those mentioned in the “Temperature measurement comparison study” section. The primary difference is that in the SNR comparison study, only the body coil is used as the transmitting coil and receiving coil to simplify the SNR calculation. This is because the SNR calculation of the phased array coil is more complicated than single channel coil and it is not necessary for us to know the SNR enhancement of the WICS coil. In addition, we only obtained T2-weighted images and but not thermal mapping because it was not necessary for SNR comparison.

### Data analysis

To obtain and evaluate temperature precision with and without using the WICS coil, averaged values and standard deviations were calculated for the square ROIs (25 pixels) on non-heating temperature maps. $\Delta \mathbf{T}$ in the non-heating temperature maps should theoretically be zero. Thus, any detected temperature variation can be interpreted as noise and distinguished easily in the non-heating temperature map.

**Figure 5:**
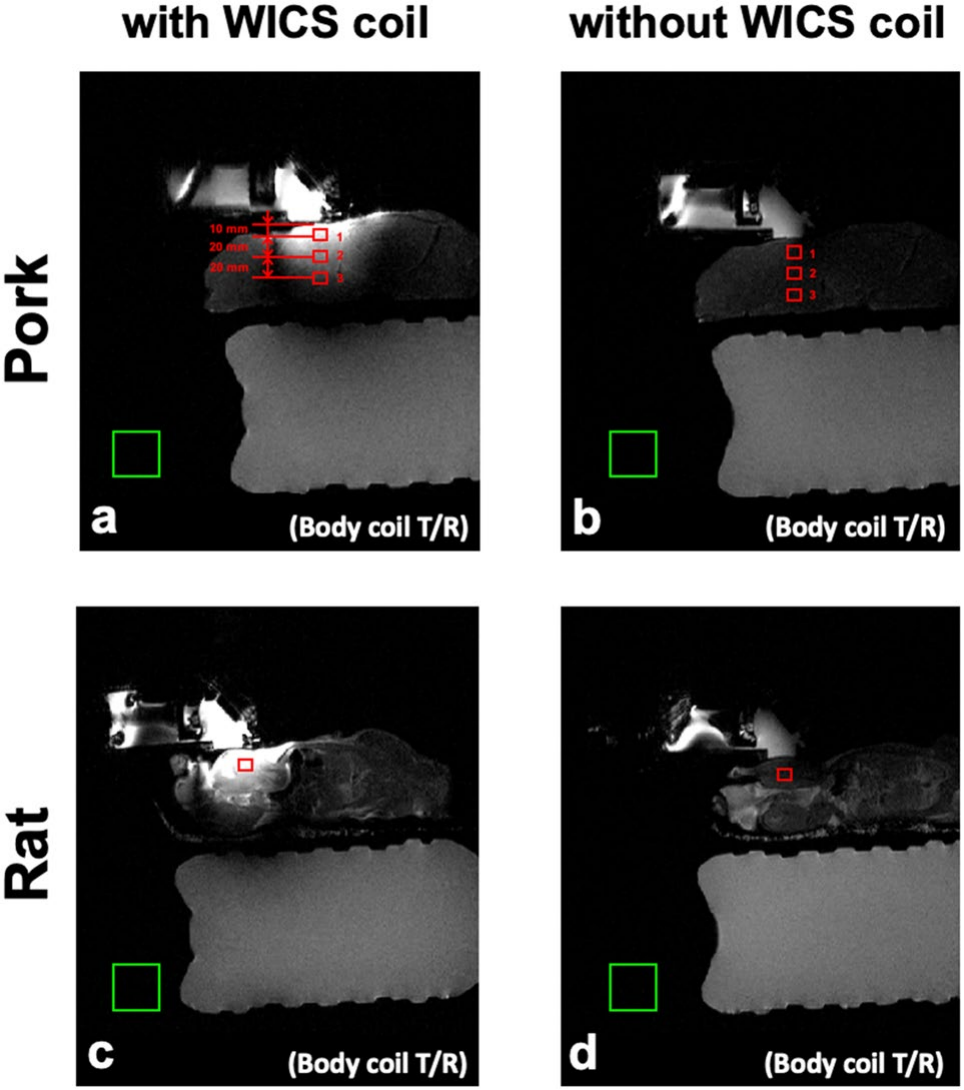
Representative T2 weighted images of rats (bottom row) and pork tenderloins (top row) obtained with (**a**, **c**) and without (**b**, **d**) WICS coil from ex vivo experiments. Each ROI contains 25 pixels for SNR measurement, as shown in red rectangles.

To evaluate the SNR enhancement from using the WICS coil, we compared the SNR obtained from T2-weighted images with and without WICS coil, using the body coil as the transmitting coil and receiving coil. The ROIs are shown in Fig. 5, and each ROI contains 25 pixels. For pork experiment, three ROIs were drawn on the T2-weighted images at different depths to compare the effect of varying distances from the WICS coil on the SNR (Note that pork tenderloin is homogeneous tissue). For rat experiment, only one ROI was drawn on T2-weighted images at the position close to the WICS coil. The SNR was calculated as the ratio of the mean ROI signal in the rat thigh or pork tenderloin to the standard deviation of the phantom signal (Eq. 4). A two-sample *t*-test was conducted to compare the SNRs of the ROIs of the T2-weighted images obtained with and without WICS coil. Comparison of SNR versus distance profile with and without WICS coil of ex vivo pork experiment was also shown.

4 $$ SNR = \frac{{S_{s} }}{{SD_{n} }} $$

where, ${S}_{s}$ (signal parameter) is the mean intensity value within the signal ROI in pork tenderloin or rat, and ${SD}_{n}$(noise parameter) is the standard deviation of intensity values within the background noise ROI.

### In vivo experiments

The in vivo animal experimental protocols were approved by the Institutional Animal Care and Use Committee of National Taiwan University. All the procedures were carried out in accordance with relevant approved guidelines and regulations. All animal protocols were performed in compliance with the ARRIVE guidelines. An adult Sprague–Dawley rat (250–350 g) were used in the in vivo experiment. Besides the anesthetization and euthanasia procedure, the experimental setup, process and MRI protocols are identical to the “ex vivo experiments” subsection but excluding the control experiment without WICS coil. The rat was placed in an anesthesia box and administered induction anesthesia by inhalation of 4% isoflurane. After setting up the rat on the MRI table, maintenance anesthesia was administered through continuous inhalation of 2.5% isoflurane via the mask cone. MR images were acquired with the WICS coil using the body coil as the transmit coil and receive coil. After the MRgHIFU ablation experiment, the rat was anesthetized by inhalation of 5% isoflurane and sacrificed by guillotine.

## Results

**Figure 6:**
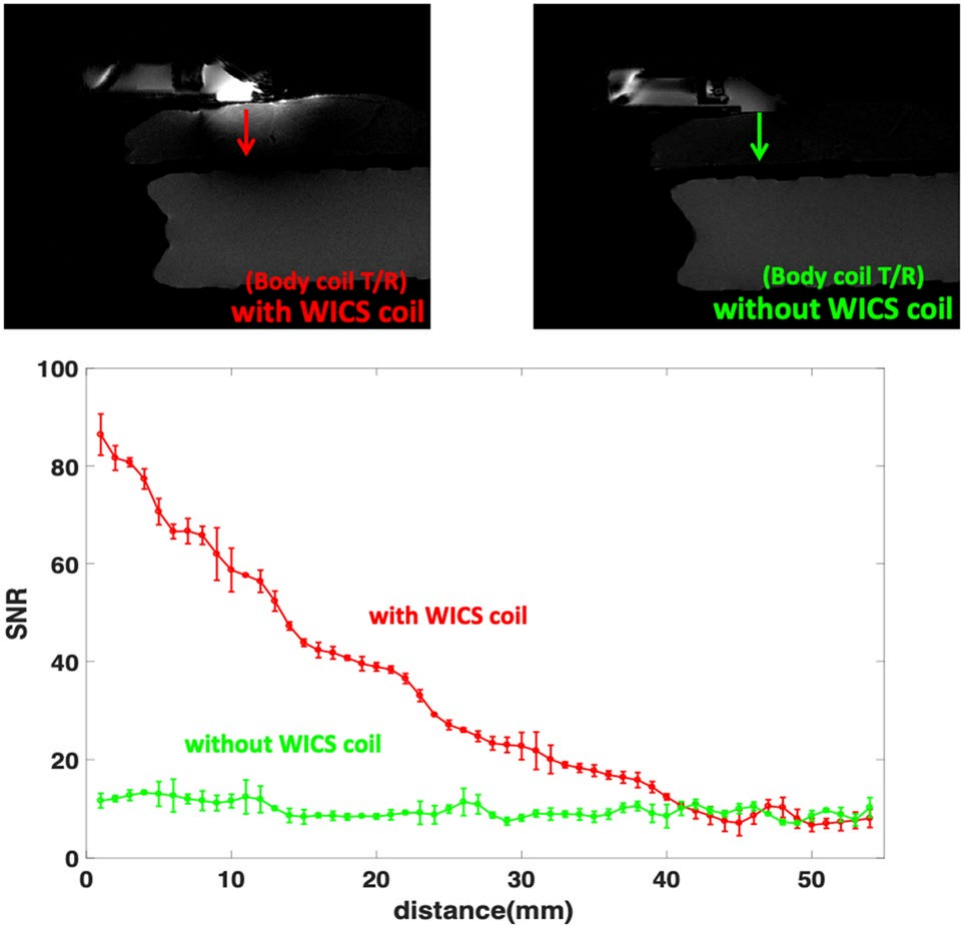
Top: The representative SNR maps with and without WICS coil in the ex vivo pork experiment. Bottom: Comparison of SNR through pork (arrow) derived from the top maps.

Figure 5 shows T2-weighted images of the rat hind thighs and pork tenderloins in the ex vivo experiments, obtained with and without WICS coil for comparison. As shown in Fig. 5, the signal intensity using the WICS coil is obviously higher when compared to using only the body coil. According to Eq. 4, higher signal intensities result in higher SNR (assuming noise parameter stays constant), and according to Eq. 3, a higher SNR of magnitude image results in a higher temperature precision. Table 1 compares the SNRs within the ROIs shown in Fig. 5. SNR measurements with versus without WICS coil differed significantly for the rat thigh and ROIs 1 and 2 of the pork experiments (*p* < 0.0001). As the distance to the WICS coil increased, the SNR decreased substantially from ROIs 1 to 3 of the pork experiment. The SNR improvement of ROI 3 is marginal because the distance from WICS coil to ROI 3 exceeds the effective region of WICS coil. Figure 6 shows the representative T2-weighted images and the SNR versus distance profile with and without WICS coil in the ex vivo pork to observe the relation of SNR improvement and the distance of WICS coil.

**Table 1:** Mean and standard deviations for SNR estimation in the ex vivo pork (*n* = 7) and rat (*n* = 5) experiments and two-sample *t*-test analysis between SNR values with and without WICS coil (body coil only).

	Sequence	WICS coil SNR	Body coil only SNR	*p* value
Rat	T_2_	113.9 ± 29.0	11.5 ± 1.3	< 0.0001
Pork	T_2_			
#ROI1		115.0 ± 31.6	15.0 ± 2.6	< 0.0001
#ROI2		47.0 ± 18.1	13.9 ± 1.9	< 0.0001
#ROI3		13.1 ± 4.3	12.2 ± 1.4	0.402

**Figure 7:**
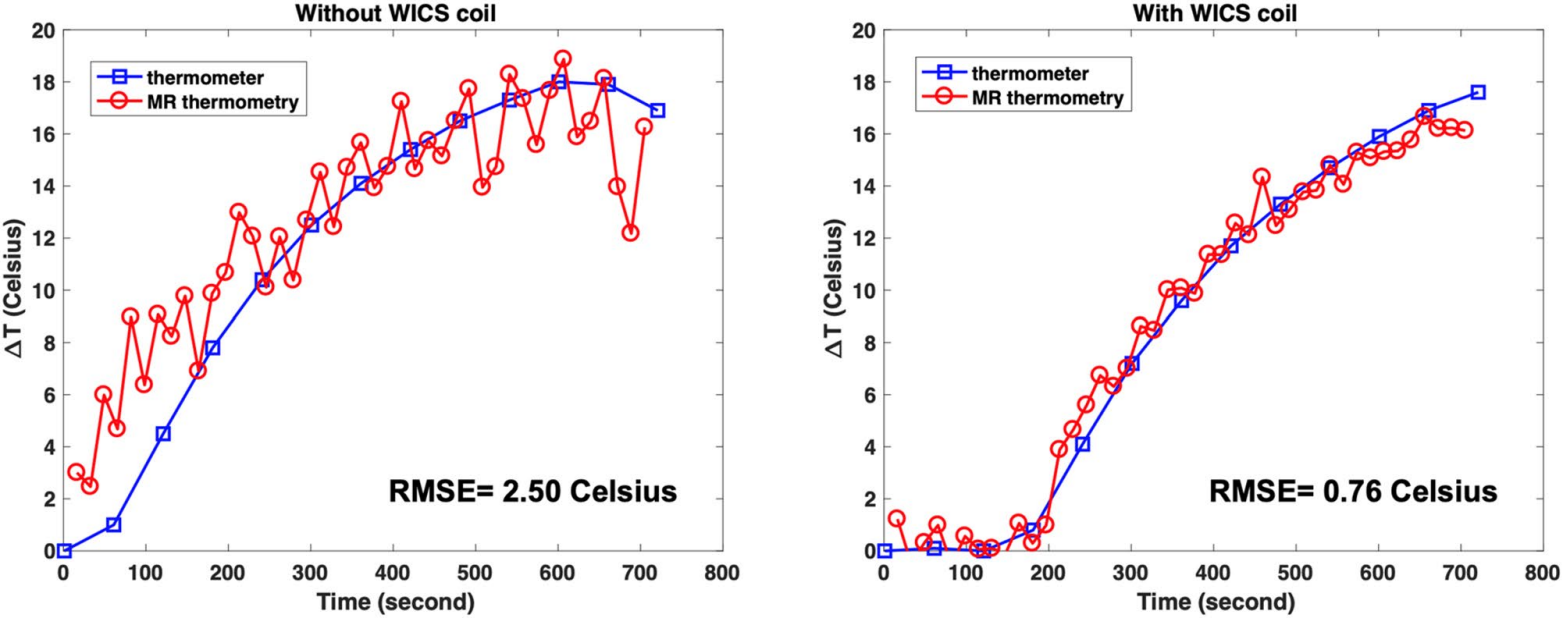
Temperature response during MR thermal mapping validation study (hot water experiment) measured simultaneously by the fiber-optic thermometer and MR thermometry scanned with and without WICS coil.

Figure 7 shows the temperature responses during the MR thermal mapping validation study, where a hot water pipe was used to heat pork tenderloin. The temperature values was measured simultaneously by the fiber optic thermometer and MR thermometry scanned with and without WICS coil. When the WICS coil was implemented, there was high consistency between MR thermometry and thermometer measurements, with temperature accuracy approximately 0.76 °C. In contrast, MR thermometry without using the WICS coil had substantial noise and temperature accuracy of approximately 2.5 °C.

**Figure 8:**
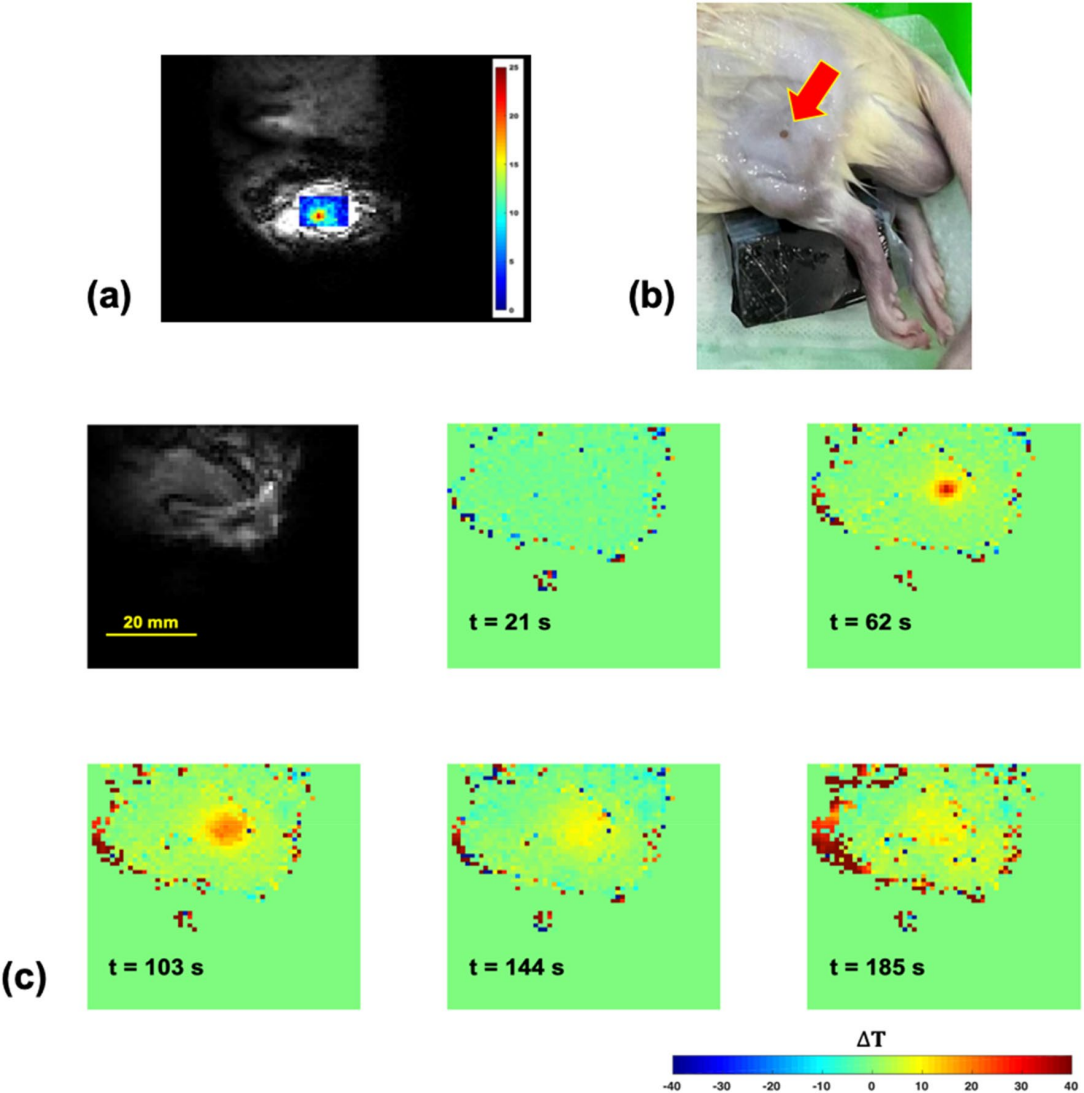
(**a**) Superimposed image of temperature map and coronal-view of the rat hind thigh at t = 82 s. (**b**) Photograph of the rat hind thigh after HIFU ablation. Red arrow indicates the lesion. (**c**) The representative magnitude image (upper left) and temperature maps obtained from ex vivo rat experiments at t = 21 s, 62 s, 103 s, 144 s and 185 s.

Figure 8(a) shows a representative superimposed image of the temperature map and coronal-view of the rat hind leg at t = 82 s in the rat HIFU experiments. Figure 8(b) shows the lesion of the rat hind leg after HIFU ablation. Figure 8(c) shows the representative temperature maps at various times, demonstrating the temperature elevation and cool-down periods in the ex vivo rat experiment.

**Figure 9:**
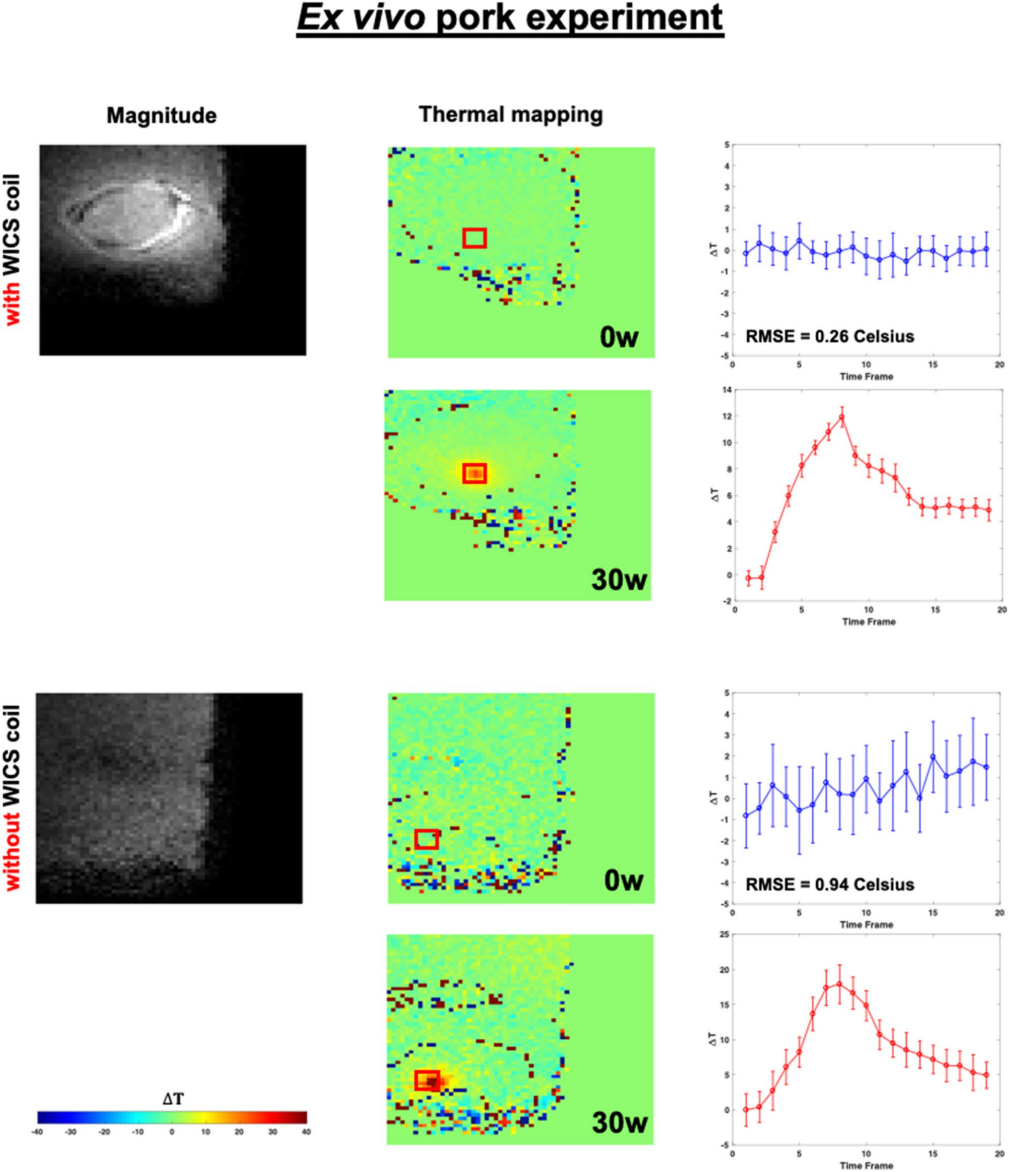
The magnitude image and the corresponding temperature map marked with the location of the ROI in the HIFU ex vivo pork experiment, with (upper) and without WICS coil (lower) for both without and with heating.

**Figure 10:**
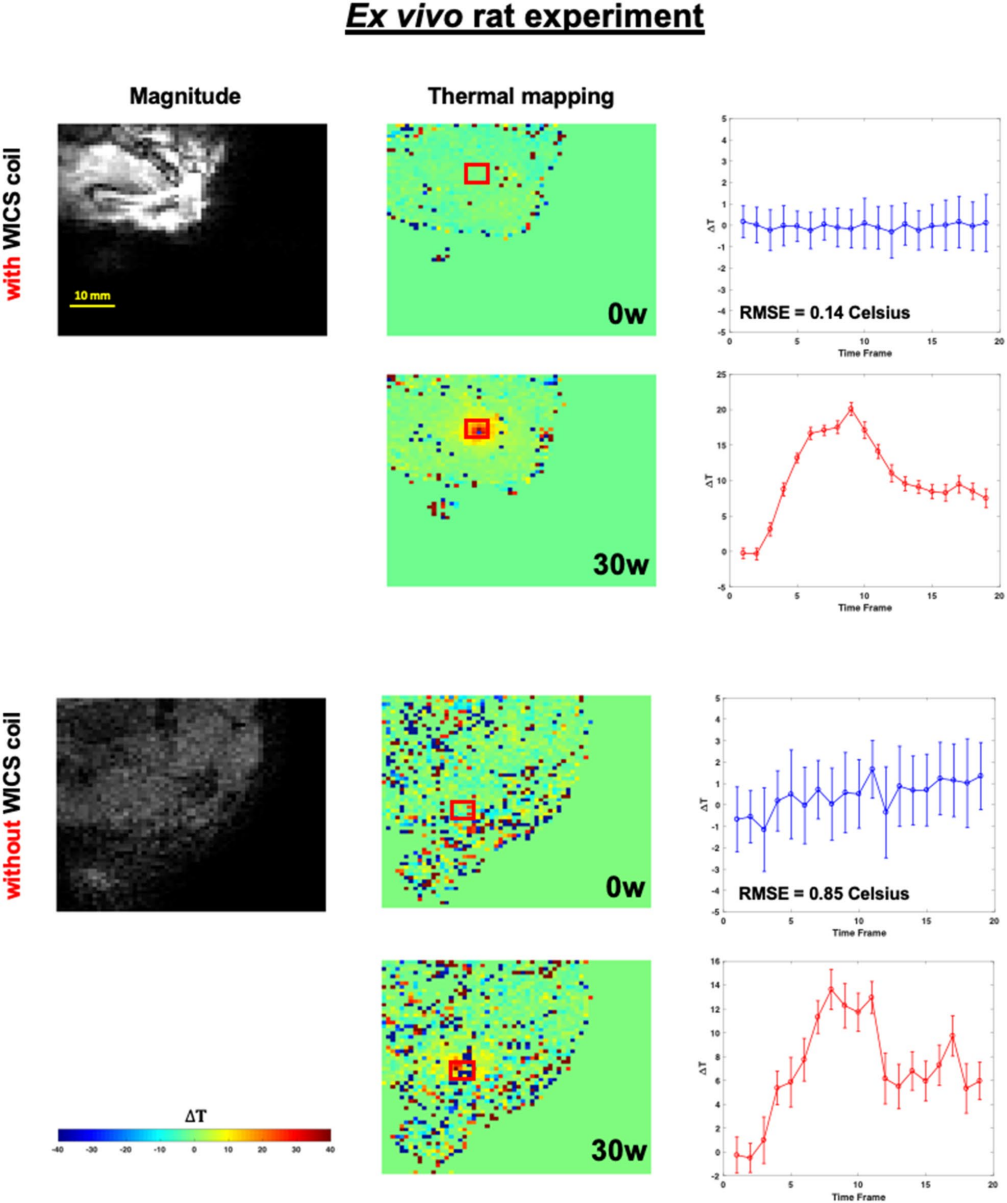
The magnitude image and the corresponding temperature map marked with the location of the ROI in the HIFU ex vivo rat experiment, with (upper) and without WICS coil (lower) for both without and with heating.

Figures 9 and 10 shows the representative temperature maps and temperature responses (mean of ROI) of the ROI in the ex vivo HIFU experiments without heating to compare temperature accuracy with and without using WICS coil. For both the ex vivo rat and pork experiments, temperature accuracy is approximately 0.26 °C and 0.14 °C when using WICS coil. In contrast, the temperature accuracy is approximately 0.94 °C and 0.85 °C in the absence of WICS coil. Figures 9 and 10 also shows the representative temperature maps and temperature responses during heating with and without WICS coil.

**Figure 11:**
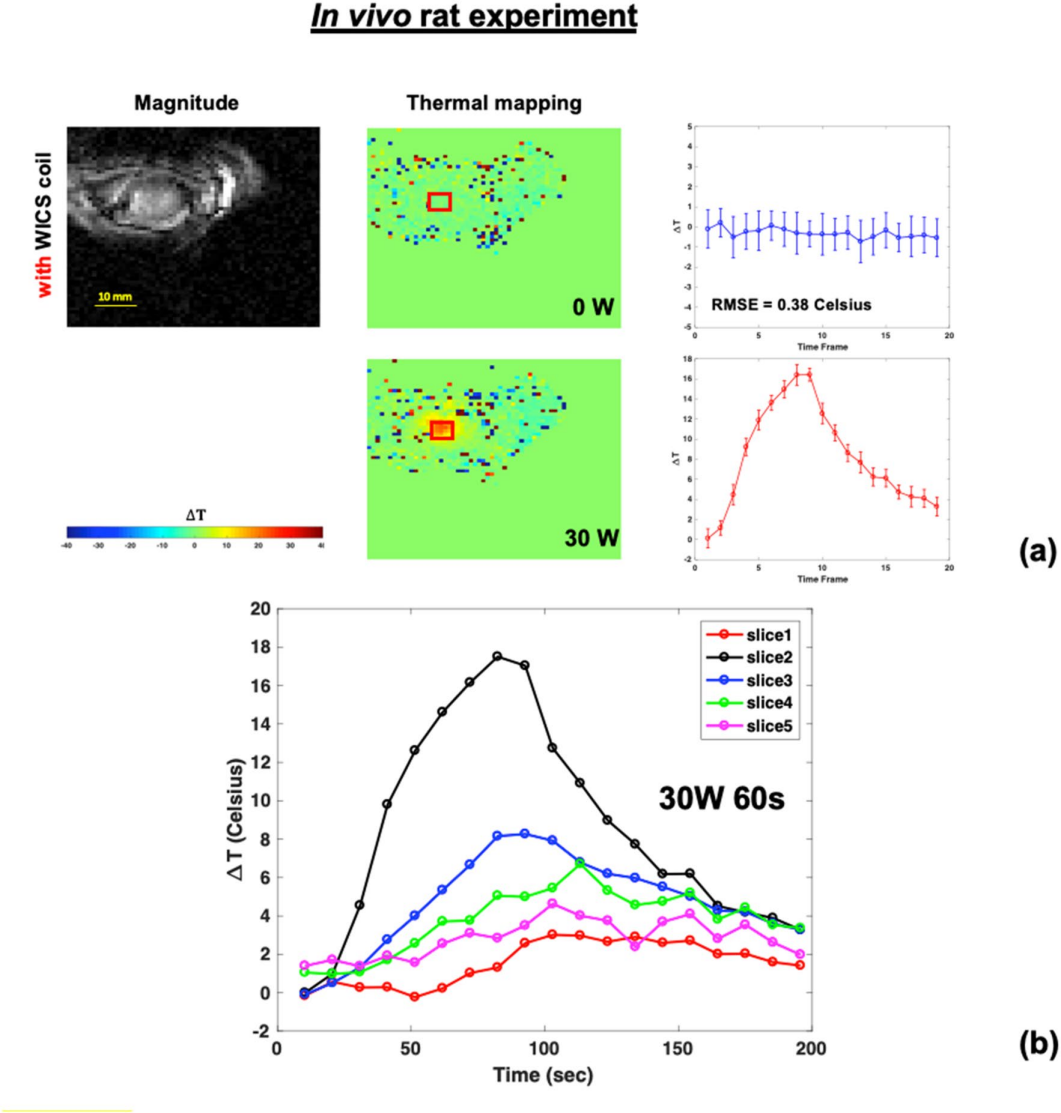
(**a**) The magnitude image and the corresponding temperature map marked with the location of ROI in the HIFU in vivo rat experiment, without (upper) and with (lower) heating. (**b**) Temperature response of in vivo experiment where focal point located in slice 2.

Figure 11(a) shows the representative temperature maps alongside the mean and standard deviation of the ROI in the in vivo HIFU experiments without heating to observe temperature accuracy with WICS coil. Temperature accuracy is approximately 0.5 °C. Figure 11(b) shows temperature responses of in vivo experiment with slice 2 as the focal point. Then temperature elevation decreases from slice 3 to slice 5.

## Discussion and conclusions

This study demonstrates the feasibility of performing small animal MRgHIFU experiments on clinical 3 T MRI with custom-made WICS coil and reflecting HIFU transducer. Our results show precise temperature measurements in ex vivo pork tenderloin, ex vivo rat and in vivo rat. This approach offers an alternative option for those who aim to employ preclinical MRgHIFU experiments on clinical MRI systems by improving the signal quality and temperature accuracy.

The major limitation of conducting preclinical studies on clinical MRI systems is the insufficient SNR when imaging small animals. To improve SNR, the WICS coil design can be directly integrated into any existing clinical MRI systems (with proper frequency tuning and matching) without any hardware modifications. In our case, the WICS coil improves temperature accuracy from 0.85 to 0.14 °C, which is adequate for monitoring the MRgHIFU treatment. Although SNR can be improved by custom-made surface coil^10^, this has to be purchased separately and is consequently more expensive and hard to implement. Moreover, the RF cable would impede the repositioning of the HIFU transducer. In contrast, the WICS coil is compact, easier to fabricate, easier to modify size to optimize for the specific application and easier to implement into any brand of clinical MRI systems directly. Most importantly, the absence of RF cables in the WICS coil allows it to be used in MRgHIFU experiments.

The reflecting HIFU transducer is a novel design which places a 45° glass in front of a piezoelectric ceramic to reflect and change the direction of the HIFU power. Gear structures behind the piezoelectric ceramic can change its location, which then changes the depth of the HIFU focal zone. This saves space because changes in the limited *y*-direction space are transformed into *z*-direction. The *y*-direction of MRIs are always limited due to obstruction from the head coil. Although depth variation is not necessary in this study, this novel design can be used in future human studies requiring depth variation.

As demonstrated in this study, the use of WICS coil effectively increases SNR. The radius of the WICS coil may affect its efficiency. When the distance from WICS coil to ROI is larger than radius (ROI 3), SNR improvement is marginal. Thus, it is essential to design an WICS coil appropriately based on the target subject and tissue depth. If the radius of the WICS coil is not appropriately designed for the depth of the target tissue, the SNR improvement will be moderate.

The results in temperature responses show the highest temperature elevation in slice 2 and then temperature elevation decreased from slice 3 to slice 5. Temperature elevation of slice 1 and slice 3 should be close in theory, because they are equally distant from the focal point (slice 2); however, the HIFU transducer is placed directly on top of slice1 leading to the circulating water dissipating the heat of slice 1.

This study focused on demonstrating the enhancement of SNR and temperature accuracy by using WICS coil, which can be mostly addressed in ex vivo rat experiments (head coil Rx). For in vivo rat experiment (body coil Rx), the purpose was to test the feasibility of utilization of WICS coil for in vivo scans. Our data shows both the ex vivo and in vivo results achieved temperature accuracy within 0.5 °C, suggesting the feasibility of WICS coil on in vivo scans. Furthermore, in vivo study uses body coil as receive coil directly. With WICS coil can still achieve the expected temperature accuracy. Although the feasibility has been well demonstrated, further studies of using the WICS coil on clinical MRI systems are of great interests, such as hemorrhage detecting, BBB permeability changing and myocardial changing of rats on clinical MRI.

The choice of receiver coil when using WICS coil is important. Although the temperature accuracy within effective region of WICS coil when using the body coil Rx is comparable to the head coil Rx, we still suggest using specific coil (such as head coil) as receiver coil to perform such experiment instead of using body coil as receiver coil directly. Because using specific coils with sufficient filling factor to acquire images can obtained better image quality outside the effective region of WICS coil. Further, we have to note that single loop receiver coil would not compatible with WICS coil (especially when its size is similar to/smaller than WICS coil) because the general single loop coil didn’t use decoupling approach to isolate itself and the amount of coupling of WICS coil would interfere the single loop receiver coil. Thus, phase array coil (such as head coil) is an appropriate choice when using WICS coil because phased array usually incorporates multiple decoupling approaches^32^ to improve the isolation for each channel and further isolation for WICS coil.

In conclusion, we have demonstrated the feasibility of conducting MRgHIFU ablation on clinical MRI systems for preclinical researches. In particular, the WICS coil design optimizes SNR and further achieves acceptable temperature accuracy, which is within the general temperature accuracy in MR thermometry^33–35^. Further studies applying the WICS coil to preclinical applications on clinical MRI systems are needed to validate the capability of WICS coil design.

## Data Availability

The data used to support the findings of this study is available from the first author upon reasonable request.
